# The Role that Biobanks Can Play in Driving Animal-Free Biomedical Research

**DOI:** 10.1017/erm.2026.10051

**Published:** 2026-05-14

**Authors:** Valerie Speirs, Alison Walker

**Affiliations:** 1 University of Aberdeen, UK; 2 Independent Cancer Patients’ Voice, London, UK

**Keywords:** biobanks, new approach methodology, non-animal modelling, translational research, human-specific

## Abstract

**Background:**

In 2025, transformative and transnational plans to phase out animal testing and reduce reliance on animal use in research settings were introduced.

**Methods:**

Steps towards realising these plans can be addressed by using human tissues and metadata obtained from biobanks. Biobanks securely and ethically collect, process, store, manage and distribute human biological tissues donated by patients for biomedical research purposes.

**Results:**

Biobanks have developed at pace over the last decade, expanding their repertoires to meet increasingly complex demands from researchers.

**Conclusions:**

This article outlines how the power of biobanks can be leveraged to drive innovative animal-free biomedical research, including the patient perspective.

## Introduction

Animals have been used extensively in biomedical research for centuries, neatly summarised in a historical timeline (Ref. 1). For much of this time, animal use was largely unregulated. Change was initiated in 1959 when the concept of humane experimental research was introduced as a way of controlling how animals were used in research, taking into consideration reduction, replacement and refinement. This became known as the 3Rs, which is a now widely accepted ethical framework that guides the use of animals for scientific research (Ref. 2). While the 3Rs have provided structured guiding principles, some argue that a greater emphasis should now be placed on replacing the use of animals in biomedical research (Ref. 3). Yet, animal use continues, albeit subject to adherence to strict ethical guidelines, regulations and transparent reporting (Refs 4, 5). Typically, animal use in research, usually mice, seeks to advance human health. This includes uncovering biological mechanisms, modelling disease processes and pre-clinical testing of the efficacy and safety of potential new treatments. These types of experiments have defined endpoints that are approved by ethics committees in advance to balance scientific goals with animal welfare. The development of transgenic animals, which bear patient-relevant mutations, has allowed the study of specific disease-related traits across various pathologies (Refs 6, 7, 8, 9). Of 2.64 million scientific procedures performed on live animals in the UK in 2024, 1.43 million were for experimental purposes, with nearly 70% of these conducted in rodents (Ref. 10). The overarching goal of these types of studies has been to understand and improve human health. But times are changing. Translational barriers from rodent studies to human trials are increasingly recognised and scientists are starting to promote human tissue-based alternatives (Ref. 11). More advanced *in vitro* and *in silico* technologies have been developed to accommodate this and are becoming drivers of a changing landscape. Financial constraints faced by many higher educational institutes are impacting their ability to maintain animal research facilities, acting as additional drivers towards adopting human-relevant, non-animal research methods, further emphasising the importance of biobanks. This has been reinforced further by announcements from the UK and US governments outlining transformative plans to phase out animal testing and reduce reliance on animals in research settings. Here, we outline how biobanks can lead this transformation.

## Development and evolution of biobanks

Biobanks are defined as organised collections of human biological samples, including tissues, cells, blood and blood derivatives. Their role is to securely and ethically collect, process, store, manage and disseminate such biological samples and associated metadata, e.g. donor, clinical and histopathological, for research purposes. Biobanks evolved from individual collections of tissues, often generated by surgeons with an interest in a particular disease type and who had access to surplus tissue generated through medical procedures. These were often seen as private collections used exclusively to drive an individual’s personal research (Ref. 12). The late Maggie Wilcox, widely regarded as the founder of cancer advocacy in the UK (Ref. 13) often recalled her disappointment at not being asked if her tissue could be used in scientific research after her breast cancer surgery. At the time of her diagnosis in 1997, biobanking as we know it today was in its infancy and tissue that was surplus to diagnosis was not usually retained for research. Consequently, she became a strong advocate of biobank development, particularly the Breast Cancer Now Biobank (Ref. 14) and was a member of its Tissue Access Committee for many years. Nowadays, Patient Public Involvement and Engagement (PPIE) is embedded in many biobanks, helping biobanking to evolve from purely scientific resources into those that put patients with lived experience of the disease in question at the heart, so that research becomes more inclusive, impactful and reflective of patient needs (Ref. 15).

More stringent regulations for the collection, storage and use of human tissues, including the UK’s Human Tissue Act (HTA) 2004 (Ref. 16), were gradually introduced, leading to greater transparency in tissue collection and storage and improved accessibility for researchers. Patients donating tissues to biobanks do this altruistically, following their explicit written informed consent and only if surplus tissue remains after a clinical diagnosis has been made. Hence, it follows that there should be greater transparency in the governance of the donated samples and associated data. This change has resulted in the evolution of biobanking into a specialist discipline that aims to accelerate scientific knowledge and improve healthcare.

In the early days of biobanking, scientists were often grateful for a small piece of tissue, which typically came directly from surgery without any associated metadata. Nowadays, scientists can request not only fresh, frozen and formalin fixed paraffin embedded tissues, but also blood derivatives, including serum, plasma and circulating tumour cells, whole slide digital tissue images and accompanying donor, clinical and histopathological metadata. Consequently, biobanks have expanded their repertoire to meet increasingly complex demands from researchers, with many extending beyond provision of basic clinicopathological data to include clinical follow-up, types of therapies, body mass index, parity, etc. Often, this comes at a cost to researchers as these data need to be pulled from clinical records, which takes time and effort. Minimum Information About BIobank data Sharing (MIABIS) standards were developed in 2012 to promote consistency in data collection across biobanks (Ref. 17) and have since been updated by the Biobanking and Biomolecular Resources Research Infrastructure-European Research Infrastructure Consortium, with the goal of enabling integration and comparison of data from different biobanks (Refs 18, 19).

Some biobanks are tissue-specific and even tissue-type-specific e.g. the Breast Cancer Now Biobank (BCNB) in the UK (Ref. 14) and the Australian Breast Cancer Tissue Bank (Ref. 20) focus on the provision of tissue from breast cancer patients (Ref. 21) while the Komen Tissue Bank in the US provides normal breast tissues from women and, more recently, men (Ref. 22). The BCNB additionally provides a cell culture programme allowing scientists to access tissue explants and primary cultures to advance their research (Ref. 14). This biobank pioneered a data return policy (Ref. 21) which is now commonplace for other biobanks, allowing researchers to mine a wealth of information derived from the tissues that have been donated by patients. Because many of the tissues held in biobanks may have been used across a range of different applications, this approach allows biobanks to build up increasingly detailed molecular and genomic profiles from these tissues, adding considerable value to tissue donations.

## How biobanks can reduce reliance on animals in biomedical research

When researching human disease, it makes biological sense to use human tissue to investigate a human condition. This is where biobanks have a considerable edge, as they offer human tissues from diverse human populations who live in real-world environments. Such diversity is difficult to replicate in animal models, which are typically young, inbred, housed in sterile settings and fed on uniform diets. Indeed, transitioning towards human-centric models to reduce the reliance on animal models is now a high priority for major research funding organisations. In 2024 and 2025, various national and government organisations set aside substantial resources to address this specifically (Refs 11, 23). Biobanks also played a vital role in Cancer Research UK’s Stratified Medicine Programme (Refs 24, 25) and in the 100,000 Genomes project (Ref. 26). These initiatives enabled the collection and storage of tissues that were used to deliver genomic testing at scale, identifying novel pathogenic variants in cancer and rare diseases (Refs 27, 28, 29). Even the most advanced of genetically engineered mouse models would likely have been unable to achieve this at the scale delivered by these two programmes.

Most biobanks have websites or can be found on special directories, improving the visibility of the types of samples available to scientists, in essence providing a ‘shop window’ of information for researchers. There are global (Ref. 30) and European (Ref. 31) directories, and the Tissue Directory and Coordination Centre (Ref. 32) covers UK-based biobanks. Country-specific biobanks e.g. Finland (Ref. 33), Scotland (Ref. 34), Wales (Ref. 35) and Australia (Ref. 20) also exist. Some commercial organisations also provide human tissues for research purposes, although they often come with limited metadata compared to that sourced directly from biobanks that are academic led. The £42M UK-led TRANSFORM trial incorporates a biobanking element, allowing researchers to gather and mine multi-omic data at an unrivalled scale in prostate cancer (Ref. 36). A non-exhaustive list of different sources of human tissue that can be obtained from biobanks is shown in Table 1. Tissue obtained from some of these biobanks has contributed to breakthroughs in human-derived molecular research. Molecular mechanisms surrounding the activity of sodium channels in mediating breast cancer metastasis have been identified using tissues from the Breast Cancer Now Biobank (Ref. 37). The Pancreas Genome Phenome Atlas, which is the bioinformatics platform of the Pancreatic Cancer Research Fund Tissue Bank, has enabled the translation of molecular insights obtained from tissues and data from pancreatic cancer patients into tools to enable prognosis, patient stratification and therapeutic decision-making (Ref. 38).

**Table 1.** Non-exhaustive list of specialist and general biobanks supplying various human tissue types for biomedical research Table 1. long description.

Biobank	Description	Location	URL
BBMRI-ERIC	Catalogue of EU biobanks with information on their sample collections.	EU	https://directory.bbmri-eric.eu/ERIC/directory/#/catalogue
BioIVT	Collection of human tissues with associated biofluids	Worldwide	https://bioivt.com/tissue–2
Breast Cancer Now Biobank	Breast cancer tissue, cells and blood samples	UK	https://breastcancernow.org/our-research/research-centres-and-projects/our-biobank
Charlie Teo Foundation Brain Tumour Bank	Tissue, live cells, DNA and blood to be used for brain cancer research	Australia	https://charlieteofoundation.org.au/biobanking-for-the-future/
Children’s Cancer and Leukaemia Group Tissue Bank	Collection of tumour tissue and biological material for research into paediatric and adolescent type solid cancers	UK	https://www.ncl.ac.uk/biobank/collections/cclgtb/
Komen Tissue Bank	Collection of normal breast tissue from healthy female and male volunteers	USA	https://cancer.iu.edu/ktb/index.html
London Neurodegenerative Diseases Brain Bank	Tissue samples from neurodegenerative and psychiatric disorders	UK	https://www.kcl.ac.uk/neuroscience/facilities/brain-bank
Pancreatic Cancer Research Fund Tissue Bank	Blood, urine and saliva samples from pancreatic cancer patients	UK	https://www.pcrf.org.uk/pancreatic-cancer-research/pancreatic-cancer-research-fund-tissue-bank/
PATH Biobank	Breast cancer tissues and blood samples	Germany	https://path-biobank.org/index.php/en/
Precision for medicine	Catalogue of clinically annotated oncology biosamples in various formats	Worldwide	https://www.precisionformedicine.com/
TriStar Technology Group	Catalogue of human biological samples in various formats	USA	https://tristargroup.us/
UK Brain Banks Network	Post-mortem brain and central nervous system tissue for diagnosis and research	UK	https://ukbbn.brainsfordementiaresearch.org/public/en/
UKCRC Tissue Directory and Coordination Centre	Register of tissue collections covering multiple disease types	UK	https://biobankinguk.org/

While biobanks have facilitated easier access to tissue for researchers to enable molecular discovery research, it is acknowledged that material and data transfer agreements should be in place before tissue can be provided and that legislation pertinent to specific countries needs to be observed to enable cross-border sharing of samples and data. Moreover, this can often be a lengthy and time-consuming process that can delay experimental progress. The various ways in which biobanking can contribute towards reducing and, in some cases, replacing animals in biomedical research are discussed below.

## Using human tissues and cells in different experimental platforms

### Omics

Many contemporary omics studies have used human tissue samples obtained from biobanks to study both normal and diseased tissue. Examples include scRNA-seq, which has been applied to healthy breast tissue obtained from two separate biobanks to create single-cell maps that have provided insights into the origin of human breast cancer (Refs 39, 40). Spatial transcriptomics of colorectal cancer and synchronous liver metastases has identified pathways that drive tumour progression on an individual patient basis (Ref. 41). Glioblastoma tissues acquired from a specialist neurobiobank identified that cell interactions with tumour cells and the tumour microenvironment enabled therapeutic resistance (Ref. 42). Transcriptomic analysis of biobanked post mortem human brain tissue from individuals with Alzheimer’s identified molecular pathways that may be targeted to reduce cognitive decline (Ref. 43) and analysing methylomic variation across multiple regions of post-mortem human brain tissue to identify epigenetic signatures associated with schizophrenia (Ref. 44). These new insights could not be achieved using animal models.

### In vitro models

Human tissues can also be used to create more precise *in vitro* models of human diseases that better recapitulate human disease pathophysiology, drug metabolism, immune response and gene regulation. Unlike animal models, results generated from these *in vitro* models are more likely to have greater predictive ability of what will happen in patients. These models range from simple systems using patient tissue or individual cell types to more complex 3D models.

One of the oldest and simplest techniques is tissue explants, where fragments of tissue are placed in chemically defined culture medium and maintained *in vitro.* The use of human tissue explants was described originally around the 1950s (Ref. 45) and while these still have a use today (Ref. 46), the methodology has evolved into using more refined precision cut tissue slices, typically around 250 μm thick, which maintain tissue integrity, complexity and heterogeneity (Ref. 47). Uses of precision-cut tissue slices include the study of acute and chronic lung disease (Ref. 48), kidney fibrosis (Ref. 49), cancer (Ref. 50), systemic sclerosis (Ref. 51) and the antagonistic roles of oleic and palmitic acids in pancreatic fibrosis (Ref. 52). While handling and analysis of these tissue slices can be challenging, in a breast cancer model, biomarkers of proliferation and apoptosis could be evaluated immunohistochemically to quantify the effects of endocrine and chemotherapy (Ref. 53).

Organoid models can be established directly from multiple human tissues. These models are defined as self-organising tissue sub-units that recapitulate their organ of origin. Over the last decade or so, the generation and use of patient-derived organoids (PDOs) derived from various pathologies has risen exponentially, including those from the brain (Ref. 54) and various cancer types (Refs 55, 56, 57). Colorectal cancer is one of the most extensively studied cancers using PDOs and has better predictive ability of clinical outcome than biomarker panels (Refs 58, 59). This has also been demonstrated in pancreatic, breast and ovarian cancer (Refs 60, 61, 62). In all of these cancer types, drug responses observed in PDOs closely mirrored clinical outcomes, indicating the power of PDOs for personalised medicine.

Since PDOs capture the complexity and heterogeneity associated with human tissue, this has enabled mechanistic studies as well as evaluating drug responses (Ref. 63). Indeed, so-called ‘living’ PDO biobanks have even been established, typically from gastrointestinal tissues, including paired primary colorectal and metastatic liver (Ref. 64) and pancreatic cancer (Ref. 65) and other pathologies, including Crohn’s disease (Ref. 66). Some PDOs are also available commercially, giving researchers direct access to these cutting-edge models, without the need to generate these in house, which can be a costly and time-consuming endeavour.

Primary cell populations can be generated from different tissue types and from different donors. Often, primary cultures can outperform traditional human cell lines that have been used for decades to model different pathologies *in vitro.* Although well characterised, readily available and easy to grow and maintain in culture, cell lines lack the variability that primary cells can offer. For example, the human hepatic cell line Hep2G lacks CYP450 activity, which is critical for performing liver-specific functions, but is retained in human HepaRG cells, an alternative organotypic co-culture that integrates hepatocytes and cholangiocytes (Ref. 67). Bespoke human cells, including normal and tumour epithelial cells, fibroblasts and immune cells can be requested from some biobanks and used to develop *ex vivo* human tissue models which can reduce animal use. Examples include ductal carcinoma *in situ* (DCIS), a model of pre-invasive breast cancer, where breast myoepithelial, luminal cells and fibroblasts were sourced from a biobank to create a DCIS model to use for cancer initiation studies (Refs 68, 69). These models offer fully humanised alternatives to the Mouse INtraDuctal (MIND) xenograft model, which involves intraductal injection of patient-derived primary DCIS cells in immunodeficient mice (Ref. 70). It is also possible to isolate and co-culture primary human salivary gland epithelial cells with T cells obtained from primary Sjögren’s syndrome patients, which have been used to test the efficacy of Rituximab in Sjögren’s syndrome (Ref. 71). Using single cell types, such as human pulmonary fibroblasts, offers the potential to enable fibroblast-targeting strategies for cancer therapy (Ref. 72]. Fibroblasts are an important component of the stromal microenvironment in cancer and when implanted into mice, human stromal cells present with the tumour tissue are gradually lost, being replaced with stromal cells from the murine host (Ref. 73), highlighting deficiencies associated with animal models when studying stromal cell biology.

### Organ on a chip and 3D bioprinting

Human tissues and cells obtained from biobanks can also be applied to various contemporary methodologies, e.g. organ-on-a-chip (OOC) microfluidic devices (Ref. 74) and in 3D bioprinting (Ref. 75). OOC devices use microfluidics to create stable chemical gradients, model dynamic mechanical stresses carefully mimicking the cellular microenvironment seen in humans. These typically consist of an array of tiny channels, membranes, and chambers, which can be lined with different cell types to model different organ functions. OOC has enabled innovative approaches, e.g. combining primary intestinal epithelial cells with innate immune cells to study immune response when the gut lining is infected by *Toxoplasma gondii*, a widespread pathogen that affects billions of people globally (Ref. 76) and a humanised model of wound healing to explore how ongoing neutrophil-driven inflammation can damage surrounding tissues and sustain chronic inflammatory responses, contributing to non-healing wounds (Ref. 77). Importantly, OOC requires only small amounts of cells and tissue, which is advantageous for biobanks. Some diagnostic specimens are small in nature, e.g. in lung cancer and due to the successes of screening programmes in breast and bowel cancer, patients are now presenting with ever smaller lesions. With the clinical priority always on diagnosis, this often leaves very little available for scientific research, meaning that scientists must turn towards innovative methods like those offered by microfluidic approaches. 3D bioprinting has been inspired by conventional 3D printing and involves the patterned layering of living cells, alongside biomaterials and/or growth factors to generate 3D structures that closely mimic human tissues or organs (Ref. 78). While still at a relatively early stage of development, current thinking proposes the use of 3D bioprinting to study a diverse range of pathologies, including Parkinson’s disease (Ref. 79), skin cancer (Ref. 80), lung disease (Ref. 81), and in tissue engineering and regenerative medicine (Ref. 82).These rapidly expanding methods are ideally positioned to make human tissues the default and could transform biomedical research by reducing animal use. Before widespread and effective implementation, there is a need to develop standards to ensure reproducibility and comparability of results. This has been recognised for both OOC and 3D bioprinting (Refs 83, 84).

## Artificial intelligence (AI) and machine learning (ML)

Innovations in AI and ML technologies now extend to tissue-based studies. The application of multimodal deep learning tools to interrogate haematoxylin and eosin (H&E)-stained tissue sections, which are routinely available in histopathology archives and can be transformed into digital whole slide images (WSIs), has witnessed rapid expansion. Clinically meaningful information that is invisible to the human eye on H&E images can be obtained from these, particularly in cancer. For example, biomarker prediction and outcome predictions, immune and inflammatory gene signatures and identification of clinically actionable genetic alterations can be detected in a range of different cancer types (Refs 85, 86, 87, 88). WSIs can also be used for 3D computational modelling (Ref. 89). These approaches are often iterative, requiring large numbers of WSIs and associated longitudinal metadata, e.g. outcomes and endpoints at a level of scale that can only be provided by biobanks. Biobanks have been pivotal in securing sufficient numbers of the rare male breast cancer, where five were accessed to obtain sufficient numbers of samples to enable the application of machine learning algorithms, which showed that attention-based multiple instance learning models trained on female breast cancer were ineffective when applied to male breast cancer (Ref. 90).

*In silico* disease modelling can be realised by using bioinformatics tools to improve our knowledge of the molecular mechanisms underlying various diseases and how this can be leveraged towards potential advances in treatments. By integrating genomic, transcriptomic, and proteomic data, bioinformatic approaches can help researchers identify critical pathways driving disease progression, therapeutic resistance, and interactions with the tumour microenvironment across various cancer types (Ref. 91).

Omics approaches using new and/or existing data derived from human tissue samples can be mined to provide comprehensive maps of cell type-specific signatures across various pathologies. The power of applying multimodal AI-driven analysis to biobanked lung and breast tissues has been demonstrated recently in a proof-of-principle study from OPTIMA, a consortium of leading European institutions that have come together to revolutionise cancer treatment through AI. The study showed the potential of multimodal AI-driven analysis integrating clinical, imaging, and omics data to enable personalised treatment, reduce invasive procedures and improve patient monitoring (Ref. 92). It has even been proposed that AI could be used to improve the quality and efficiency of developing human tissue-based models (Ref. 93), such as the organoid models described above. The ability of ML algorithms to analyse large quantities of existing data could be harnessed, e.g. to identify the best type and composition of supporting matrix and how to create this more efficiently, identify necessary growth factors and relevant external stimuli, as well as refining cell culture conditions (Ref. 94). A recent study of squamous cell head and neck cancer described a PDO platform integrating AI-driven digital pathology and genomics to assess drug response (Ref. 95), offering a scalable framework for translational cancer research. Taking this a step further, it has been suggested that tissues themselves could be generated *in silico*, using data being produced from ongoing spatial analyses (Ref. 96). So-called digital twins, which serve as a digital model of an individual’s biological tissue, are gaining momentum in healthcare due to their ability to integrate vast amounts of patient data (Ref. 97). However, this approach still relies on using real-world data generated from human tissues, placing biobanks at the heart of this.

## Data return enables biobank enrichment

Contemporary biobanks provide not only tissues, but the implementation of data return policies from tissue-based studies means that they represent rich data resources (Ref. 21). The ability of some biobanks to link longitudinal biospecimens and multimodal data, such as electronic health records, -omics and imaging data (Ref. 98) will only expand this, informing personalised medicine approaches and the creation of digital twins (Ref. 97). Interrogation of longitudinal datasets has potential in predicting outcomes of drug exposure, disease progression, or gene interactions, which cannot be achieved using animal models.

While unlikely to benefit current donors, data enrichment and sharing could generate information that may be transformative for future generations. This is often articulated by patients when they make a tissue donation (personal communication). Using patient data requires consideration of privacy, consent and data governance. Because biobanks already have ethical approvals in place, these are normally taken care of in the form of data sharing agreements, but biobank recipients should always double-check before taking receipt of samples and/or data.

## The patient perspective

A powerful driver in moving away from using animals in research is patients and the public. From our own work, we know that patients donating to biobanks often object to their tissues being used in experiments that require animals. There is an expectation that donated tissue will be used in ways that are relevant to human health and carried out to high ethical standards. Many patients are increasingly aware that results from animal studies do not always translate well to people, and some have concerns about animal welfare. As a result, patients often prefer their donated tissues to be used in human-based, animal-free research, where this is scientifically appropriate. The option to limit or opt out of animal use through the consent process is therefore important and helps patients feel confident that their wishes are respected.

For patients, donating tissue to a biobank is an act of trust and generosity. Donation often takes place at a difficult time, typically around diagnosis, surgery or treatment. Patients usually donate not for personal benefit, but because they want their experience of disease to help improve understanding, treatments and outcomes for future patients. Biobanks play a key role in building and maintaining patient trust. Clear consent processes, strong governance, such as participation in advisory groups or tissue access committees, help ensure that research decisions reflect patient values as well as scientific priorities.

Patients also value transparency about how their donated tissues are used. Although donors do not expect direct benefit, they appreciate knowing that their donation is contributing to meaningful research. Communication about research outcomes and the use of data generated from donated tissues helps reinforce the value of their donations and encourages continued public support for biobanking.

The growing use of human tissues to develop advanced laboratory models, computational approaches and AI represents a positive change in biomedical research. These approaches focus on human biology and often provide more relevant insights than animal models. Supporting biobanks and animal-free research, therefore, aligns scientific progress with patient values and expectations and helps ensure that tissue donations are used in ways that most directly benefit human health.

## Challenges

Scientists are protocol-driven and often reluctant to move away from tried and tested methods. This is understandable in a climate where research funding is hard fought, and career progression can be at stake. Furthermore, journal reviewers and editors often insist that data is validated in an animal model. However, perceived barriers to the development and implementation of human tissue models are gradually being relieved as the use of human tissue models starts to be implemented and adopted more widely. Indeed, a recent opinion piece championed the transition towards so-called non-animal models (NAMs), including the platforms described in this article, highlighting their superior ability to model human physiology, improve translational accuracy and minimise ethical issues (Ref. 11).

Although biobanks strive to collect and make available a wide range of tissue types, even the most well-resourced biobanks cannot provide everything. Not all centres have biobanks, which can deny patients the opportunity to donate their tissues even if they wish to do so. Furthermore, patient participation cannot always be guaranteed even in centres with biobanking infrastructure. An ideal scenario would be the complete integration of biobanking into the diagnostic pathway. There is evidence that this can work when there are sufficient resources available and goodwill from clinical teams (Ref. 99) but question marks remain for more widespread implementation.

Rarer samples are often at a premium for scientists, meaning that biobank tissue access committees sometimes must prioritise the studies that they feel are most likely to generate information that will benefit patients. The patient voice on these committees is valuable in helping reach a decision. Prioritisation of studies is not an issue for some animal models of disease, where almost unlimited supplies can be generated through breeding programmes. However, there is often poor translatability of animal studies, with failure rates of over 90% often quoted for drug discovery pipelines (Ref. 100). Moreover, this counters the 3Rs, which seek to Replace, Reduce and Refine the use of animals in research (Ref. 2) with the landscape now shifting much more in favour of replacement strategies (Ref. 3).

## Conclusion

Science and technology are evolving at a pace. Discovery research focused on human tissue and data, and not animal models, is similarly gaining momentum, driven by international initiatives that are prioritising the development and implementation of NAMs (Ref. 23). As demonstrated throughout this review, the use of tissues provided by biobanks is already contributing to breakthroughs in human-derived molecular research, improving translational relevance to benefit human health. As NAMs advance and become more universally adopted, this trend will surely continue. Biobanks are ideally positioned to accelerate this change, such that using human tissues and data becomes the default position in driving scientific innovation, advancing molecular and personalised medicine and reducing animal use to meet contemporary research needs and those of the patient.

## Table 1. Long description

Column headers from left to right are Biobank, Description, Location, and URL. The first row lists B B M R I dash E R I C, described as a catalogue of E U biobanks with sample collection information, located in E U, URL is https://directory dot b b m r i dash e r i c dot e u forward slash E R I C forward slash directory forward slash number sign forward slash catalogue. The second row is Bio I V T, offering human tissues with biofluids, worldwide, URL is https://bioivt dot com forward slash tissue dash 2. The third row is Breast Cancer Now Biobank, providing breast cancer tissue, cells, and blood samples, U K, URL is https://breastcancernow dot org forward slash our dash research forward slash research dash centres dash and dash projects forward slash our dash biobank. The fourth row is Charlie Teo Foundation Brain Tumour Bank, supplying tissue, live cells, D N A, and blood for brain cancer research, Australia, URL is https://charlieteofoundation dot org dot au forward slash biobanking dash for dash the dash future forward slash. The fifth row is Children’s Cancer and Leukaemia Group Tissue Bank, with tumour tissue and biological material for paediatric and adolescent solid cancers, U K, URL is https://www dot n c l dot a c dot u k forward slash biobank forward slash collections forward slash c c l g t b forward slash. The sixth row is Komen Tissue Bank, collecting normal breast tissue from healthy female and male volunteers, U S A, URL is https://cancer dot i u dot e d u forward slash k t b forward slash index dot html. The seventh row is London Neurodegenerative Diseases Brain Bank, with tissue samples from neurodegenerative and psychiatric disorders, U K, URL is https://www dot k c l dot a c dot u k forward slash neuroscience forward slash facilities forward slash brain dash bank. The eighth row is Pancreatic Cancer Research Fund Tissue Bank, providing blood, urine, and saliva samples from pancreatic cancer patients, U K, URL is https://www dot p c r f dot org dot u k forward slash pancreatic dash cancer dash research forward slash pancreatic dash cancer dash research dash fund dash tissue dash bank forward slash. The ninth row is P A T H Biobank, with breast cancer tissues and blood samples, Germany, URL is https://path dash biobank dot org forward slash index dot php forward slash en forward slash. The tenth row is Precision for medicine, a catalogue of clinically annotated oncology biosamples in various formats, worldwide, URL is https://www dot precisionformedicine dot com forward slash. The eleventh row is TriStar Technology Group, catalogue of human biological samples in various formats, U S A, URL is https://tristargroup dot u s forward slash. The twelfth row is U K Brain Banks Network, post-mortem brain and central nervous system tissue for diagnosis and research, U K, URL is https://ukbbn dot brainsfordementiaresearch dot org forward slash public forward slash en forward slash. The thirteenth row is U K C R C Tissue Directory and Coordination Centre, register of tissue collections covering multiple disease types, U K, URL is https://biobankinguk dot org forward slash.

Navigate back to Table 1..
